# Paradoxical tuberculosis-associated immune reconstitution inflammatory syndrome in initiating ART among HIV-Infected patients in China-risk factors and management

**DOI:** 10.1186/s12879-023-08897-3

**Published:** 2024-01-02

**Authors:** Honghong Yang, Qian Liu, Yushan Wu, Kun He, Qin Zeng, Min Liu

**Affiliations:** Division of Infectious Diseases, Chongqing Public Health Medical Center, 109 Baoyu Road, Shapingba District, Chongqing, 400036 China

**Keywords:** Tuberculosis(TB), human immunodeficiency virus (HIV), Immune reconstitution inflammatory syndrome (IRIS), Integrase strand transfer inhibitor (INSTI)

## Abstract

**Background:**

China is a country burdened with a high incidence of both tuberculosis (TB) and HIV, Paradoxical tuberculosis-associated immune reconstitution inflammatory syndrome (TB-IRIS) is an important early complication in TB and HIV co-infected patients, but data from China are limited. Additionally, as an integrase strand transfer inhibitor (INSTI)-based antiretroviral therapy (ART) regimen becomes the first-line treatment, concerns have arisen regarding the potential increase in the incidence of paradoxical TB-IRIS. Nevertheless, the existing data are inconclusive and contradictory.

**Methods:**

We conducted a retrospective study at Chongqing Public Health Clinical Center from January 2018 to December 2021. We collected demographic and clinical data of HIV/TB co-infected patients who initiated ART. We described the patient characteristics, identified predictors for TB-IRIS, and determined clinical outcomes. The Statistical Package for Social Science (SPSS 25) was used to analyse the data. Continuous variables were compared using Student’s t-test or rank sum test. Counting data were compared using the chi-square test or Fisher’s exact test. The variables with statistical significance in the univariate analysis were added to the binary logistic regression. A p-value less than 0.05 was considered statistically significant.

**Results:**

A total of 384 patients co-infected with naive HIV and pulmonary TB (PTB) who were given ATT and ART combination were included. 72 patients (18.8%) developed paradoxical TB-IRIS with a median of 15 (12, 21) days after initiating ART. Baseline age ≤ 40years, CD4 + T-cell counts ≤ 50cells/µL, HIV viral load ≥ 500,000 copies/mL were found to be significantly associated with development of paradoxical TB-IRIS. Mortality rates were similar in the TB-IRIS (n = 5, 6.9%) group and non-TB-IRIS (n = 13, 4.2%) group. Interestingly, CD4^+^ T-cell counts recovery post-ART was significant higher in the TB-IRIS group when compared to the non-TB-IRIS group at the end of 24 weeks (P = 0.004), as well as at 48 weeks (P = 0.015). In addition, we consider that INSTI- based ART regimen do not increased the risk of Paradoxical TB-IRIS.

**Conclusion:**

Paradoxical TB-IRIS, while often leading to clinical deterioration and hospitalization, is generally manageable. It appears to have a positive impact on the recovery of CD4 + T-cell counts over time. Importantly, our data suggest that INSTI-based ART regimens do not elevate the risk of TB-IRIS. Thus, paradoxical TB-IRIS should not be considered an impediment to initiating ART in adults with advanced immunodeficiency, except in the case of tuberculous meningitis (TBM).

## Introduction

Tuberculosis (TB) and human immunodeficiency virus (HIV) stand as two of the most pressing global public health concerns [1]. TB remains not only one of the most prevalent opportunistic infections (OIs) among people living with HIV (PLHIV) but also the leading cause of mortality in this population [2]. Early initiation of antitubercular treatment (ATT) and antiretroviral therapy (ART) is widely recognized as a crucial strategy for enhancing survival [3]. However, the optimal timing for initiating ART in PLHIV with TB infection remains a subject of debate, particularly among those with tuberculous meningitis (TBM). On one hand, some studies suggest that early ART initiation can be particularly beneficial for patients with advanced HIV [4–6]. On the other hand, the expedited restoration of the immune system associated with early ART initiation carries a significantly heightened risk of paradoxical tuberculosis-associated immune reconstitution inflammatory syndrome (TB-IRIS) [7]. Notably, it has been reported that paradoxical TB-IRIS leads to hospitalization in approximately 25% of cases and contributes to IRIS-related deaths in the range of 2–12% [3, 5]. The fear of managing paradoxical TB-IRIS is a common reason behind the delay in initiating ART.

Paradoxical TB-IRIS represents a transient yet occasionally severe local and systemic inflammatory response, often resulting in a temporary exacerbation or deterioration of TB symptoms, signs, and/or radiographic findings [9]. The incidence of TB-IRIS varies, ranging from 8 to 54%, with a higher incidence among those with advanced HIV [10–12]. Despite its clinical significance, the pathophysiology and immunopathology of paradoxical TB-IRIS remain not fully understood. Several clinical risk factors, including lower CD4 + T-cell counts, CD4/CD8 ratio, higher viral load (VL), and shorter intervals between ATT and ART, have been associated with the development of paradoxical TB-IRIS [13, 14]. However, it’s noteworthy that not all PLHIV with TB under similar conditions develop paradoxical TB-IRIS [15–17]. Furthermore, in the current era, the introduction of integrase strand transfer inhibitor (INSTI)-based ART regimens, known for their rapid recovery of CD4 + T-cell counts and VL reduction, raises questions regarding the potential increase in the incidence of paradoxical TB-IRIS. However, the existing data on this topic are limited, and comprehensive large-scale cohort studies are lacking.

China, as the country with the third-largest TB burden, faces these challenges as well. Paradoxical TB-IRIS in China, particularly in southwest regions such as Chongqing, where both TB and HIV prevalence are notable [18–21], has not been comprehensively investigated. Whether the overall incidence of paradoxical TB-IRIS is higher in this region remains unclear. Therefore, this study collated and analyzed reported cases of paradoxical TB-IRIS at Chongqing Public Health Medical Center (CPHMC) from January 2018 to December 2021. The study’s objectives encompass understanding the epidemiological characteristics of TB-IRIS, identifying risk factors among TB/HIV co-infected patients, assessing treatment outcomes, and evaluating long-term immunological effects. Additionally, the study sought to determine whether INSTI-based regimens could heighten the risk of TB-IRIS and whether adjustments to the timing of ART initiation are warranted for patients co-infected with TB and HIV, particularly when selecting the INSTI-based regimen.

## Methods

### Study design

We conducted a retrospective cohort study involving patients co-infected with HIV and pulmonary TB (PTB) who received ART at CPHMC from January 2018 to December 2021. Data for this study were extracted from electronic medical records and encompassed baseline demographic information, including symptoms, gender, age, routine blood counts, biochemical test results, CD4 + T-cell counts, HIV VL, and ART regimens. We also collected data on CD4 + T-cell counts and VL at 24 and 48 weeks after the initiation of ART.

### Definitions

Inclusion criteria for this study were as follows: (1) Age above 18 years, (2) HIV infection diagnosed in accordance with the guidelines for HIV/AIDS diagnosis and treatment in China (2021) [22], (3) Confirmation of active PTB, which included positive sputum smear, positive mycobacterial culture, or positive molecular testing, with or without extrapulmonary TB (EPTB). (4) Receipt of ATT, and (5) Initiation of ART following medical advice. Patients co-infected with PTB and HIV were categorized into two groups: TB-IRIS and non-TB-IRIS, based on the occurrence of IRIS.

Patients with PTB or EPTB received a standard ATT regimen in accordance with national guidelines. All patient care was overseen by three dedicated TB physicians. Diagnosis of paradoxical TB-IRIS adhered to the International Network for the Study of HIV-associated IRIS (INSHI) 2008 consensus definition [23], which required the fulfillment of additional criteria: a 0.5-1 log10 decline in HIV VL values with or without an associated increase in CD4 + T-cell count for a definite IRIS diagnosis. For a definitive diagnosis, at least one major criterion or two minor clinical criteria were necessary. Major criteria included: (1) New or enlarging lymph nodes, cold abscesses, or other focal tissue involvement, (2) New or worsening radiological features of tuberculosis, (3) New or worsening TBM, (4) New or worsening serositis. Minor criteria included: (1) New or worsening constitutional symptoms, (2) New or worsening respiratory symptoms, (3) New or worsening abdominal pain accompanied by peritonitis, hepatomegaly, splenomegaly, or abdominal adenopathy. Each suspected case of paradoxical TB-IRIS was reviewed by two members of the clinical coordination team.

### Analysis

We conducted all statistical analyses using Statistical Package for the Social Sciences (SPSS) software, version 25 (SPSS Inc., Chicago, Illinois, USA). Normally distributed continuous data were summarized with means and standard deviations (SDs) and compared using Student’s t-test. Continuous variables without normal distribution were presented as median and the 25th to 75th interquartile range (IQR) and compared using rank sum test. Categorical variables were presented as frequencies or percentages and compared using the chi-square test or Fisher’s exact test as appropriate. Potential risk factors for TB-IRIS, including pre-ART demographic variables, HIV parameters, and the time interval between ATT and ART initiation, were evaluated through simple logistic regression, followed by adjusted logistic regression. We estimated odds ratios and 95% confidence intervals (CI). A p-value less than 0.05 was considered statistically significant.

## Results

### Characteristics of the study population

**Fig. 1:**
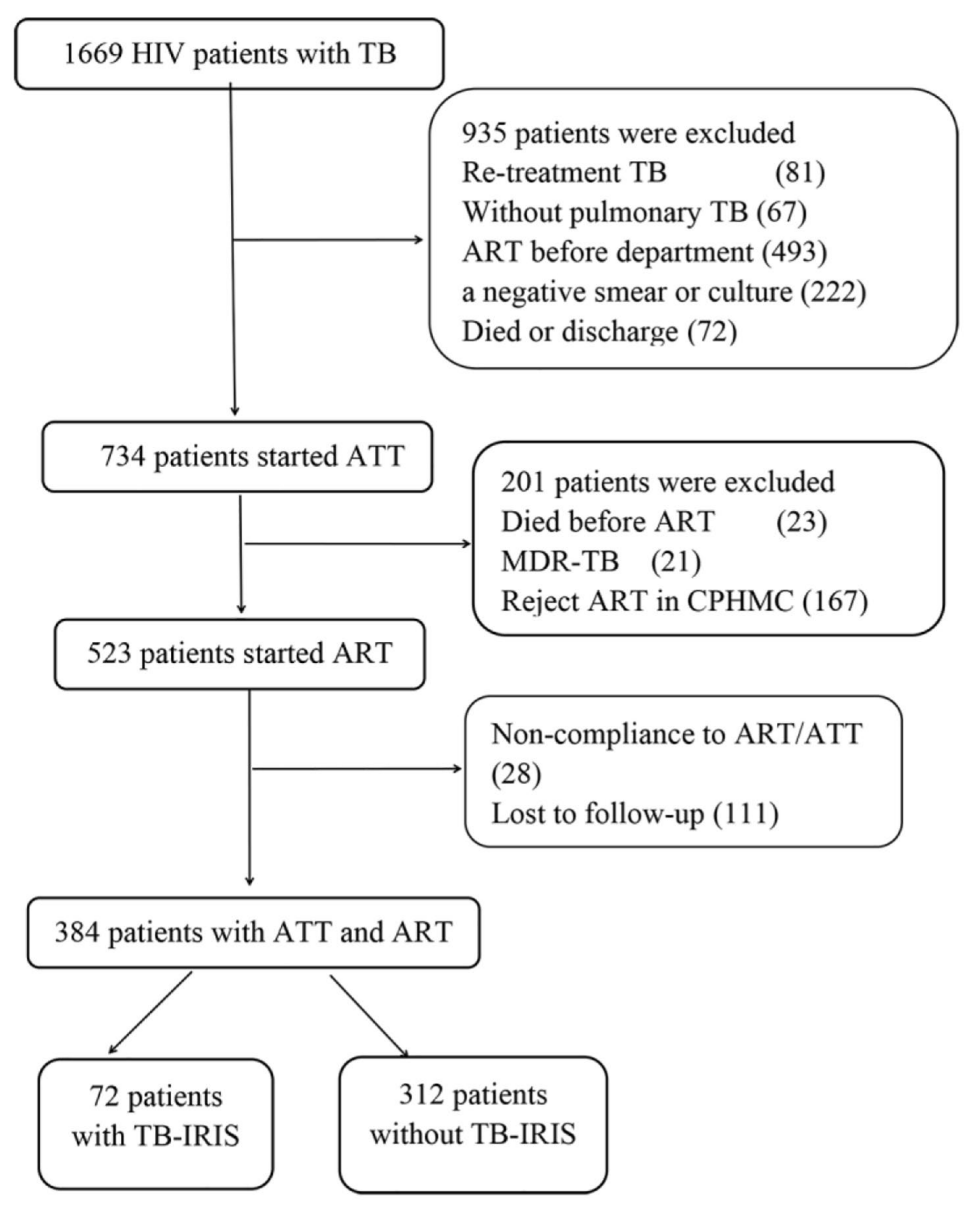
Study enrollment schema

From January 2018 to December 2021, a total of 7192 PLHIV were admitted to CPHMC. Among these, 1669 HIV patients (23.2%) were diagnosed with PTB in the department. After excluding patients with non-compliance to ART or ATT and those lost to follow-up, a total of 384 treatment-naive patients co-infected with HIV and PTB who received both ATT and ART were enrolled in the study. The patient enrollment process is illustrated in Fig. 1.

The majority of the 384 patients were male (84.6%, 325), with a median age of 50 years (interquartile range (IQR) 38–61 years). Heterosexual transmission was the primary route of HIV transmission, accounting for 90.9% (349/384). Of these patients, 32.0% (123/384) had 1–4 forms of EPTB. The most common sites of EPTB involvement were tuberculous pleurisy (17.7%, 68/384), followed by tuberculous lymphadenitis (13.5%, 52/384), tuberculous meningitis (TBM) (10.7%, 41/384), intestinal TB (6.3%, 24/384), and other forms of EPTB (7.8%, 30/384).

### Incidence of IRIS events

Among the 384 patients, 72 individuals (18.8%) experienced paradoxical TB-IRIS events within 24 weeks of initiating ART. The most common clinical manifestations of paradoxical TB-IRIS were fever (72.2%, 52/72), followed by worsening cough and expectoration (30.6%, 22/72), new-onset headache (8.3%, 6/72), deteriorating consciousness (6.9%, 5/72), new-onset superficial lymph node enlargement (6.9%, 5/72), and worsening cold abscess (2.8%, 2/72). The frequently observed radiological manifestations of paradoxical TB-IRIS included new-onset pulmonary infiltrates (55.6%, 40/72), new-onset intra-abdominal or intra-thoracic lymphadenopathy, new-onset pleural or intra-abdominal effusion, and worsening intracranial mass lesions (Table 1).

**Table 1:** Manifestations in 72 patients with tuberculosis-associated immune reconstitution inflammatory syndrome

Manifestation	No. of patients (%)
Clinical	
New-onset fever	52/72 (72.2%)
Aggravating of cough and expectoration	22/72 (30.6%)
New-onset headache	6/72 (8.3%)
Deteriorating consciousness	5/72 (6.9%)
New-onset superficial lymph node enlargement	5/72 (6.9%)
Worsening cold abscess	2/72 (2.8%)
Radiological	
New-onset pulmonary infiltrates	40/72 (55.6%)
New-onset intra-abdominal or intra-thoacic lymphadenopathy	5/72 (6.9%)
New-onset pleural or intra-abdominal effusion	4/72 (5.6%)
Worsening intracranial mass lesion	4/72 (5.6%)

### TB-IRIS risk factor analysis

The pre-ART characteristics of patients who developed paradoxical TB-IRIS versus those who did not are summarized in Table 2. In the univariate analysis, there were no significant differences between the two groups regarding gender and albumin (ALB) levels. The median time between initiating ATT and ART in the TB-IRIS group was 15 days (IQR: 12–21), ranging from 6 to 63 days, while for patients in the non-TB-IRIS group, it was 16 days (IQR: 13–21), ranging from 5 to 134 days, and the difference was not statistically significant (P = 0.495). In contrast, significant differences were observed in several baseline characteristics. The TB-IRIS group had a higher proportion of individuals aged 40 years or younger, lower hemoglobin levels (≤ 90 g/L), lower CD4 + T-cell counts (≤ 50 cells/µL), and higher VL (≥ 500,000 copies/mL) at baseline in peripheral blood. Furthermore, a greater frequency of miliary TB was detected in the TB-IRIS group compared to the non-TB-IRIS group. Notably, among the 384 patients, 176 (45.8%) initiated ART based on INSTIs, while 208 (54.2%) commenced ART based on non-INSTIs as the initial treatment. The incidence rates of TB-IRIS were 20.5% and 17.3% in the INSTI and non-INSTI groups, respectively (P = 0.431). These findings suggest that the initiation of INSTI-based treatment does not increase the risk of TB-IRIS.

**Table 2:** Baseline Predictors of Paradoxical TB-IRIS in 384 TB-HIV-Coinfected Patients Starting ART - Univariate Analysis

Parameter (numbers/percentage)	Total	TB-IRIS	Non-TB-IRIS	*P*
	n = 384	n = 72	n = 312	
Age				0.003
≤ 40 years	106(27.6%)	30(41.7%)	76(24.4%)	
IQR	32(27, 34)	34(29, 36)	31(26, 34)	
≥ 40 years	278(72.4%)	42(58.3%)	236(75.6%)	
IQR	55(49, 63)	55(49, 60)	56(49, 64)	
Male	325(84.6%)	61(84.7%)	264(84.6%)	0.982
PTB disease				
Smear positive	238(62.0%)	48(66.7%)	190(60.9%)	0.363
Culture positive	261(68.9%)	56(80%)	205(66.3%)	0.055
Molecular testing	104(27.2%)	22(26.3%)	82(26.3%)	0.462
Miliary	61(15.9%)	17(23.6%)	44(14.1%)	0.047
Extra-pulmonary tuberculosis				
Tuberculous pleurisy	68(17.7%)	15(20.8%)	53(17.0%)	0.441
Tuberculous lymphadenitis	52(13.5%)	12(16.7%)	40(12.8%)	0.390
Tuberculous meningitis	41(10.7%)	10(13.9%)	31(9.9%)	0.382
Intestinal TB	24(6.3%)	7(9.7%)	17(5.4%)	0.177
HB (≤ 90 g/L)	132(34.4%)	33(45.8%)	99(31.7%)	0.023
ALB (≤ 30 g/L)	134(34.9%)	25(34.7%)	109(34.9%)	0.973
Initial CD4^+^T-cell counts (≤ 50cells/µL )	188(49.0%)	47(65.3%)	141(45.2%)	0.002
Initial HIV VL (≥ 500,000 copies/mL)	194(50.5%)	47(65.3%)	147(47.1%)	0.005
Time between ATT and ART initiation (days)	16(13, 21)	15(12,21)	16(13, 21)	0.495
HAART regimen				0.431
INSTI- based	176	36(20.5%)	140(79.5%)	
Non- INSTI- based	208	36(17.3%)	172(82.7%)	

Abbreviations: PTB, pulmonary tuberculosis; EPTB, Extra-pulmonary tuberculosis, HB, haemoglobin; ALB, albumin; ATT, antitubercular treatment; ART antiretroviral therapy;

**Fig. 2:**
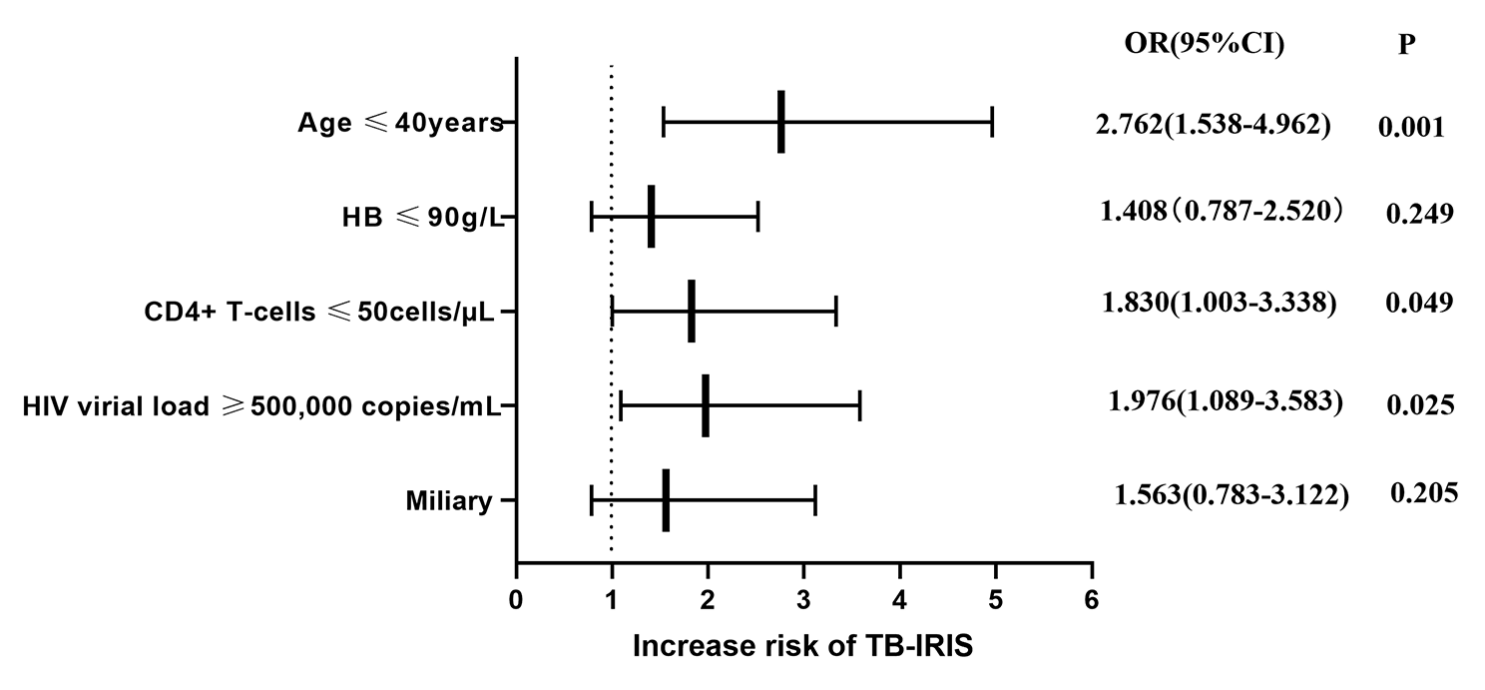
Associations between pre-ART clinical and laboratory characteristics with subsequent paradoxical TB-IRIS events. The variables that showed a significant relationship with the development of paradoxical TB-IRIS (from Table 1), that is, age, haemoglobin, baseline CD4^+^ T-cell counts, HIV VL, and miliary were included in this predictive model. Association of all variables with risk for TB-IRIS was assessed in adjusted logistic regression models. Odds ratios for values below or above threshold levels were displayed in a forest plot R—odds ratio; CI—confidence level

Using a logistic regression model, we investigated the association of pre-ART characteristics with the subsequent development of paradoxical TB-IRIS (Fig. 2). The variables that showed a significant (or trending towards significant) relationship with the development of TB-IRIS were included in this predictive model. An increased risk of developing paradoxical TB-IRIS was associated with age ≤ 40years, baseline CD4^+^ T-cell counts ≤ 50 cells/ul, baseline HIV VL ≥ 500,000 copies/mL. However, The association between paradoxical TB-IRIS and lower haemoglobin level (≤ 90 g/L), miliary were not statistically significant after adjustment for potential confounding factors.

### Management and outcome of IRIS

Among the 72 patients who developed paradoxical TB-IRIS, seven patients experienced TB-IRIS before discharge, while 64 patients required re-hospitalization due to TB-IRIS, and one patient was managed in the outpatient department. The time intervals from ART initiation to the presentation of TB-IRIS were 13 days (IQR: 9–22), ranging from 3 to 116 days. Patients experiencing TB-IRIS were initially treated with nonsteroidal anti-inflammatory drugs (NSAIDs) for the first 3–5 days if they presented with symptoms such as fever, pain, and discomfort. In cases where patients had a suboptimal response or persistent symptoms with NSAIDs, prednisolone (0.5-1 mg/kg/day) in 59 patients (59/72, 81.9%) was administered and tapered over a period of 2 to 8 weeks, depending on the severity and response. Interrupting ART was necessary for only two patients (4.9%, 2/41) due to severe TB-IRIS, while the remaining 70 patients continued ART throughout their treatment. The median time for the resolution or significant improvement of symptoms was 11 days (IQR: 6–15).

The overall mortality rate was similar between the TB-IRIS group (6.9%, n = 5) and the non-TB-IRIS group (4.2%, n = 13) during the follow-up period (P = 0.315). Of the five patients diagnosed with paradoxical TB-IRIS who died, contributing causes of death included TBM-IRIS (n = 3), multiple organ failure (n = 1), and one patient’s cause of death remained undetermined. In the non-IRIS group, 13 deaths occurred after ART initiation, with two attributed to TBM, while the cause of death for the remaining 11 patients was unclear. The mortality rate was higher in the TBM-IRIS group (30%, 3/10) compared to the TBM-IRIS group (6.5%, 2/31) (P = 0.083).

### Immune restoration post-ART

Overall, both the TB-IRIS and non-TB-IRIS groups exhibited substantial increases in CD4 + T-cell counts over the 48 weeks of follow-up. However, the TB-IRIS group demonstrated significantly higher increases in CD4 + T-cell counts compared to the non-TB-IRIS group at the end of 24 weeks (P = 0.004) and at 48 weeks (P = 0.015). There was no significant difference in the proportion of patients with undetectable HIV VL between those who experienced paradoxical TB-IRIS and those who did not, both at 24 weeks and 48 weeks after ART initiation (Table 3).

**Table 3:** Outcomes at 24, 48 weeks post-ART initiation

	Total (384)	TB-IRIS (72)	Non TB-IRIS (312)	*P*
HIV response				
HIV VL < 50 copies/mL at week 24, n (%)	248(248/332, 74.7%)	48(48/62, 77.4%)	200(200/270, 74.1%)	0.585
HIV Vl < 50 copies/mL at week 48, n (%)	281(281/331, 84.9%)	53(53/63, 84.1%)	228(228/268, 85.1%)	0.850
Increase of CD4^+^T-cell counts (cells/µL) at week 24*, median [IQR]	81(36, 122)	97(55, 130)	62(25, 117)	0.005
Increase of CD4^+^T-cell counts (cells/µL) at week 48*, median [IQR]	101(49, 170)	132(79, 201)	65(28, 115)	0.015

## Discussion

This retrospective study of 384 patients represents one of the largest cohorts reporting the prevalence, risk factors, and outcomes of paradoxical TB-IRIS in China. The significance of this study lies in the fact that the data were obtained from a region burdened by high HIV and TB prevalence. In recent years, INSTI-based ART regimens, such as dolutegravir (DTG)/bictegravir, emtricitabine, and tenofovir alafenamidem(B/F/TAF), known for their swift efficacy in reducing VL, have been widely used in newly diagnosed PLHIV in China. However, there is a scarcity of studies on TB-IRIS in this specific setting. In our study, more than 20% of hospitalized PLHIV were diagnosed with TB, and the prevalence of paradoxical TB-IRIS was 18.8%, which aligns with the 18% figure obtained in a meta-analysis [5] but is lower than the 22.6% reported in a recent study in China [11]. The prevalence of paradoxical TB-IRIS varies widely across the world, ranging from 4–54% [10–12], a variability likely attributed to factors such as clinical settings (inpatient vs. outpatient), geographic regions, and study designs.

Delay in HIV diagnosis is common in resource-limited areas, often due to individuals’ reluctance to get tested, leading to late-stage diagnosis when severe OIs are present. In our study, over 50% of patients had a CD4 + T-cell count below 50 cells/µL. We identified an association between the occurrence of paradoxical TB-IRIS and a baseline CD4 + T-cell count lower than 50 cells/µL, which is consistent with previous research [11, 24, 25]. We also found that a baseline HIV-1 VL higher than 500,000 copies/mL at ART initiation was independently associated with the risk of paradoxical TB-IRIS. Thus, PLWH with CD4 + T-cell counts below 50 cells/µL and a high baseline HIV-1 VL should be particularly vigilant regarding potential TB-IRIS. Unlike earlier studies [9, 26, 27], interval between ATT to ART was not associated with the later development of paradoxical TB-IRIS in the current study, this may be due to the relatively uniform schedule for 2 week in the time from ATT to ART. Late diagnosis is extremely severe among hospitalized PLHIV in Chongqing, it’s reported that the prevalence of advanced HIV was as high as 77.4% [19], and those usually combined with multiple pathogenic bacteria infection and multiple organ system damages, especially CNS infections, so the complicated circumstances led to a delay in the initiation of ART to approximately 2 weeks or more, however, the overall prognosis was not affected.

Age emerged as a notable risk factor for the development of paradoxical TB-IRIS, with patients younger than 40 years of age being more susceptible to experiencing TB-IRIS compared to their older counterparts. This trend can be attributed to younger patients’ relatively more robust recovery of CD4 + T-cell counts in HIV and TB co-infected adults, possibly due to relatively more preserved thymic function [28, 29].

Despite the short-term morbidity associated with IRIS and the increased risk of TB-IRIS-related hospitalization, these patients generally had good long-term outcomes. Both groups in our study displayed substantial increases in CD4 + T-cell counts over time, with patients who developed IRIS exhibiting even more pronounced improvements after 24 and 48 weeks. This suggests that patients experiencing IRIS had positive clinical outcomes, as assessed by CD4 + T-cell counts, which were at times even better than their non-IRIS counterparts, this result is similar to earlier studies [27, 30, 31].

Furthermore, we observed no association between the occurrence of paradoxical TB-IRIS and mortality in our study. The all-cause mortality in the paradoxical TB-IRIS group (6.9%) was similar to that in non-TB-IRIS patients (4.2%), aligning with the average of 7% reported in a meta-analysis [32]. The effective management of TB-IRIS was largely due to the appropriate use of corticosteroids, which are considered a first-line therapy [33, 34]. Early detection and adequate treatment significantly reduced IRIS-related mortality. The mortality rate for TBM-IRIS was notably high, reaching 30%, although this difference was not statistically significant and could be attributed to the small sample size. The timing of ART initiation for TBM also requires careful consideration.

Importantly, our study found that INSTI-based ART regimens do not increase the risk of paradoxical TB-IRIS. This contrasts with some prior studies that reported an association between ART containing INSTIs and IRIS [35, 36]. Our study suggests that the timing of ART initiation should not be delayed due to concerns about increased TB-IRIS risk when using INSTI-based regimens. Our results are consistent with a Korean retrospective study [37] and a randomized Reduction of Early Mortality (REALITY) trial conducted in sub-Saharan African countries [38]. Notably, our study exclusively focused on TB-IRIS, excluding IRIS caused by other pathogens.

Despite these insights, our study has limitations. Being retrospective, it might not have recognized some forms of paradoxical TB-IRIS, especially in patients without major clinical symptoms. Additionally, our patients had very low CD4 + T-cell counts, so the results might not be applicable to patient populations with higher CD4 + T-cell counts. Nevertheless, our study reports the largest number of IRIS cases in a single study to date, offering valuable clinical insights into paradoxical TB-IRIS.

## Conclusion

This study highlights a paradoxical TB-IRIS rate of 18.8% in China. Baseline age ≤ 40 years, CD4 + T-cell counts ≤ 50 cells/µL, and HIV VL ≥ 500,000 copies/mL are the major risk factors associated with the occurrence of paradoxical TB-IRIS. TB-IRIS is generally manageable, and patients who develop TB-IRIS tend to have positive clinical outcomes, as indicated by CD4 + T-cell counts. Importantly, INSTI-based ART regimens do not increase the risk of TB-IRIS in our study. Therefore, TB-IRIS should not hinder the initiation of ART in adults with advanced immunodeficiency, with the exception of TBM-IRIS. Further studies are needed to explore the long-term outcomes of TB-IRIS over several years and to elucidate the underlying immunological mechanisms.

## Data Availability

The datasets used and/or analysed during the current study are available from the corresponding author on reasonable request.
