# Correction: Paradoxical tuberculosis-associated immune reconstitution inflammatory syndrome in initiating ART among HIV-Infected patients in China-risk factors and management

**DOI:** 10.1186/s12879-024-09080-y

**Published:** 2024-03-01

**Authors:** Honghong Yang, Qian Liu, Yushan Wu, Kun He, Qin Zeng, Min Liu

**Affiliations:** Division of Infectious Diseases, Chongqing Public Health Medical Center, 109 Baoyu Road, Shapingba District, Chongqing, 400036 China

**Correction: BMC Infect Dis 24, 5 (2024)**

**https://doi.org/10.1186/s12879-023-08897-3**

Following publication of the original article [1], we have been notified that Fig. 2 was showing one unnecessary parameter (fever).

Originally published Fig. 2:
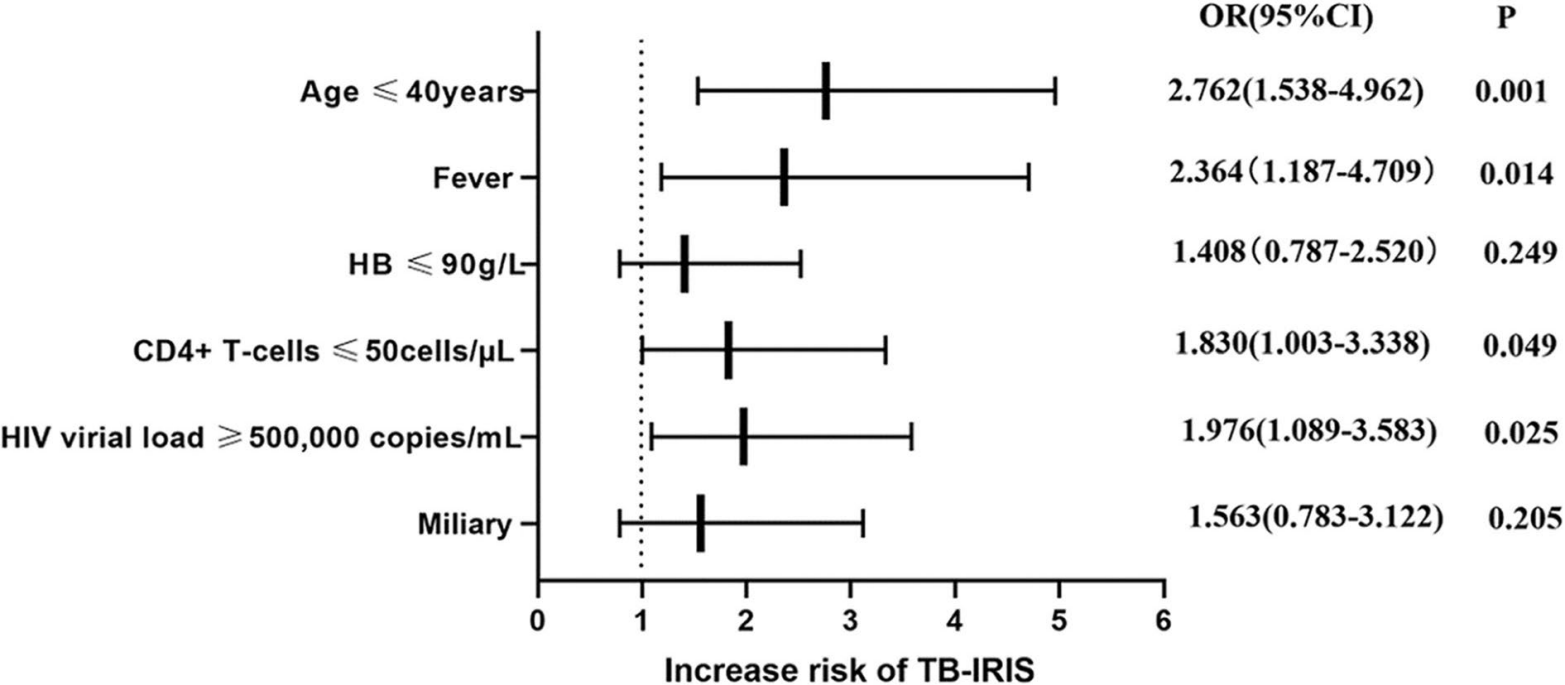


Corrected Fig. 2:

**Fig. 2:**
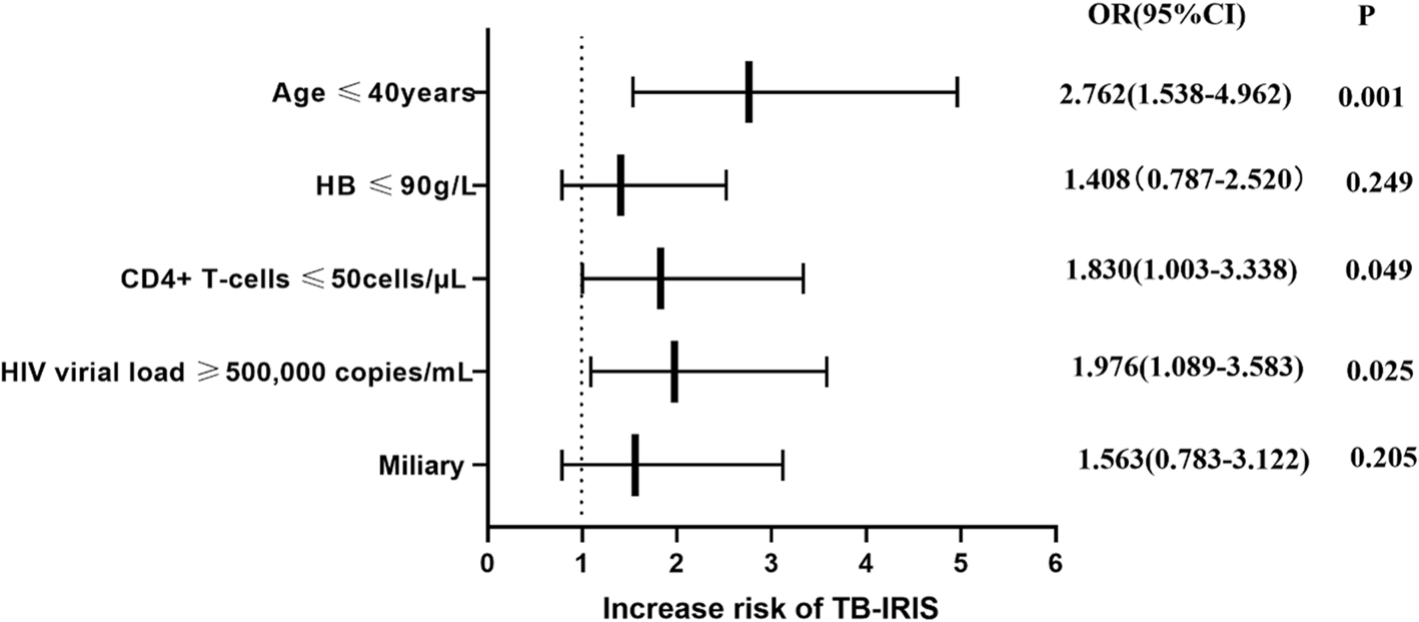
Associations between pre-ART clinical and laboratory characteristics with subsequent paradoxical TB-IRIS events. The variables that showed a significant relationship with the development of paradoxical TB-IRIS (from Table 1), that is, age, haemoglobin, baseline CD4 + T-cell counts, HIV VL, and miliary were included in this predictive model. Association of all variables with risk for TB-IRIS was assessed in adjusted logistic regression models. Odds ratios for values below or above threshold levels were displayed in a forest plot R—odds ratio; CI—confidence level

The original article has been corrected.
